# Illustrative Variant-Positive Molecular Autopsy Series: Autopsy-Led Concordance of Loeys–Dietz Spectrum Findings in Eight Florida Sudden Deaths

**DOI:** 10.3390/genes17091040

**Published:** 2026-08-29

**Authors:** Juan Fernández-Cadena, Edwin W. Naylor, Arindam Bhattacharjee

**Affiliations:** 1 Department of Pediatrics, Brigham and Women’s Hospital, Harvard Medical School, Boston, MA 02115, USA; fernandezjuan@uees.edu.ec; 2 Omics Science Laboratory, Faculty of Health Science, Universidad Espíritu Santo, Samborondón 092301, Ecuador; 3 NCGM Laboratory, Apex, NC 27502, USA; ednaylor@bellsouth.net

**Keywords:** Loeys–Dietz syndrome, *TGFBR1*, *TGFBR2*, *TGFB2*, *SMAD3*, molecular autopsy, sudden death, variant of uncertain significance, aortic dissection

## Abstract

**Background/Objectives:** Molecular autopsy increasingly identifies rare variants in heritable aortopathy and Loeys–Dietz spectrum genes among sudden-death decedents, yet classification and causal attribution remain challenging when a syndromic diagnosis was never established during life. We reviewed eight unrelated Florida medical examiner decedents ascertained by sudden or unexpected death under medical examiner jurisdiction, including laboratory-reported heterozygous missense variants spanning the Loeys–Dietz syndrome (LDS)/familial thoracic aortic aneurysm and dissection (FTAAD) gene spectrum (*TGFBR1*, *TGFBR2*, *TGFB2*, and *SMAD3*). None had a premortem clinical diagnosis of LDS; ascertainment was therefore a variant-positive molecular autopsy series, not clinically diagnosed LDS. This illustrative series of eight cases cannot estimate prevalence, diagnostic yield, penetrance, or incidental-finding frequency. **Methods:** Autopsy (±premortem) phenotype–variant concordance was graded as strong, limited, discordant, or not assessable without treating death as evidence of pathogenicity or equating laboratory detection with clinical LDS diagnosis. Grades were assigned by the authors using predefined rules, were not blinded to laboratory class, and were reconciled by consensus discussion; inter-rater reliability was not quantified. Population, computational, and ClinVar annotations, together with multi-axis molecular triangulation, were retained as supplementary context only. **Results:** Laboratory classifications were pathogenic in one case (*TGFBR1* p.Arg487Gln, Case 1), likely pathogenic in another (*TGFBR2* p.Glu428Asp, Case 3), and variants of uncertain significance (VUS) in the remaining cases. Strong phenotype–variant concordance mapped to two aortic-catastrophe decedents (Cases 1 and 3), limited concordance to two cases (Cases 4 and 5), and discordant presentations to four (Cases 2, 6, 7, and 8), including pulmonary thromboembolism without aortopathy, infant sudden death with competing cardiomyopathy-gene context, and ethanol-related death with a normal aorta (*SMAD3* Case 8). **Conclusions:** In this illustrative variant-positive series, 2/8 cases had strong autopsy-led concordance, 2/8 limited, and 4/8 discordant. Autopsy-led concordance grading distinguished two LDS-spectrum variants with strong phenotype–variant concordance for fatal ascending aortic catastrophe from six variants with limited or discordant support. Rare variants in *TGFBR1*, *TGFBR2*, *TGFB2*, or *SMAD3* should not be conflated with an LDS diagnosis or with the cause of death without concordant autopsy evidence.

## 1. Introduction

Postmortem molecular testing increasingly identifies rare variants in cardiogenetic and aortopathy genes among sudden-death decedents investigated by medical examiners, including individuals never evaluated for heritable connective tissue or aortic disease during life [1,2]. Interpretation is constrained by computational predictor discordance and frequent absence of segregation or functional data in typical molecular autopsy settings [3,4], the predominance of variants of uncertain significance (VUS) [5,6], and incomplete premortem phenotyping. Updated ACMG guidance emphasizes transparent VUS reporting and cautious clinical communication when evidence remains insufficient for definitive classification [7]. Critically, laboratory detection of a rare variant in an LDS-relevant gene does not establish a clinical diagnosis of LDS, nor does sudden death alone constitute evidence that a variant is pathogenic [2,8]. In the molecular autopsy setting, autopsy (±premortem) phenotype should therefore lead to interpretation. Concordance between documented pathology and an LDS/FTAAD-spectrum gene finding can be graded as strong, limited, discordant, or not assessable, without treating death itself as circular evidence of pathogenicity and without equating molecular detection with clinical LDS diagnosis.

Here, we report eight unrelated decedents investigated within the Florida medical examiner system in whom postmortem molecular testing identified heterozygous missense variants spanning the LDS/FTAAD TGF-β pathway spectrum (*TGFBR1*, *TGFBR2*, *TGFB2*, and *SMAD3*). Ascertainment was defined by medical examiner investigation plus reportable LDS-spectrum gene findings, not by a premortem clinical diagnosis of LDS. We apply an autopsy-led interpretive approach that foregrounds phenotype–variant concordance and treats in silico annotation and multi-axis molecular triangulation as supplementary context only. The aim of this series is to illustrate the interpretation of postmortem LDS-spectrum variants relative to autopsy findings in an already variant-positive ascertainment, without equating laboratory detection with clinical LDS diagnosis or with cause-of-death attribution, and without estimating prevalence, diagnostic yield, or penetrance.

Loeys–Dietz syndrome and related familial thoracic aortic aneurysm and dissection (FTAAD) disorders arise from dysregulated TGF-β signaling and are associated with life-threatening aortic catastrophe, often in young individuals [9,10,11]. Heterozygous pathogenic or likely-pathogenic variants in *TGFBR1* (LDS type 1), *TGFBR2* (LDS type 2), *SMAD3* (LDS type 3/aneurysm–osteoarthritis spectrum), and *TGFB2* (LDS type 4) are among the principal genes implicated in this spectrum [9,10,11,12,13,14].

## 2. Materials and Methods

### 2.1. Study Design and Case Ascertainment

This retrospective case series comprises eight unrelated decedents investigated by Florida medical examiners in whom postmortem molecular testing identified heterozygous missense variants in genes within the Loeys–Dietz syndrome (LDS)/familial thoracic aortic aneurysm and dissection (FTAAD) TGF-β pathway spectrum (*TGFBR1*, *TGFBR2*, *TGFB2*, or *SMAD3*) [9,10,11,12,13,14]. Each case has one primary LDS/FTAAD-spectrum missense variant; all other reportable findings on the same molecular report are inventoried in Supplementary Table S7 (so dual primary LDS-spectrum calls would appear there if present). Inclusion required (i) sudden or unexpected death under Florida medical examiner jurisdiction and (ii) at least one laboratory-reported LDS/FTAAD-spectrum gene variant on the postmortem molecular report. Ascertainment was therefore defined by medical examiner investigation plus reportable LDS-spectrum gene findings, not by a premortem clinical diagnosis of LDS [1,2]. Accordingly, this series should not be interpreted as eight decedents with diagnosed Loeys–Dietz syndrome, nor as a yield, prevalence, or penetrance study.

Cases are numbered 1–8 in gene/pathway order (*TGFBR1* → *TGFBR2* → *TGFB2* → *SMAD3*; Table 1). Extraction identifiers (C1–C8) are retained for laboratory-source traceability and are mapped in Supplementary Materials/case order documentation (C5 = *SMAD3* = Case 8; Case 5 is C6/*TGFB2* p.His2Arg). Variant nomenclature and laboratory classifications were taken from the postmortem molecular reports issued for each case. The series may be enriched for phenotype–variant concordance relative to unselected sudden-death molecular autopsy practice; this potential selection bias is acknowledged and precludes prevalence or diagnostic-yield inference.

### 2.2. Molecular Testing

All eight decedents underwent whole-exome sequencing (WES) as the primary assay from a single clinical laboratory vendor. Apparent heterogeneity across the series reflects differences in downstream interpretation and reporting scope rather than different underlying sequencing modalities: some cases used phenotype-/indication-driven prioritization of genes from the exome data, whereas others restricted interpretation and reporting to a predefined indication-specific gene set. Per-case clinical indication (including HPO terms when available), WES assay, downstream interpretation strategy, documented gene-analysis scope, available coverage/sequencing metrics, copy-number variant (CNV) result/reporting, and the primary LDS-spectrum finding are summarized in Supplementary Table S6, abstracted directly from the available laboratory reports and requisitions; information not documented is reported as such rather than inferred. Additional (secondary) variants reported on the same molecular autopsy reports are indicated separately (Table 2; complete inventory in Supplementary Table S7). Interpretation of additional or negative gene findings is limited to genes included in the documented analysis/reporting scope.

### 2.3. Molecular Autopsy and Clinical Data Abstraction

For each decedent, findings were abstracted from certified medical examiner records and/or laboratory clinical-indication text, including circumstances of death, relevant premortem history when documented, macroscopic findings (with emphasis on cardiovascular and aortic pathology), toxicology when reported, and the pathologist’s stated cause and manner of death when available [1,2]. Premortem echocardiography, CT or MR angiography, and systematic connective tissue phenotyping were not available in the de-identified research records and were not imputed. De-identified records did not support family history evidence and segregation analysis [8]. Consequently, a causal role for most alleles cannot be resolved without family testing, a richer premortem phenotype, or functional evidence.

### 2.4. Phenotype Review for LDS/FTAAD Spectrum

Premortem and autopsy records were reviewed for features associated with LDS/FTAAD-spectrum disease as described in GeneReviews, and documented features were compared with the gene reported on the molecular autopsy report. Premortem and autopsy features relevant to the LDS/FTAAD spectrum were reviewed using a broad feature list adapted from GeneReviews clinical descriptions (aortic dilatation/aneurysm and dissection/rupture, other arterial disease, tortuosity, craniofacial, skeletal, cutaneous, cardiac, pulmonary, allergic/inflammatory when assessable, and family history). Each feature was scored as Present, Absent, Not documented, or Not assessable from available records (Supplementary Table S5). Sudden death alone was not treated as evidence of LDS-spectrum pathogenicity.

### 2.5. Phenotype–Variant Concordance Grading

Interpretation was autopsy-led. Concordance grades were assigned by the authors (J.F.-C. and A.B.) from de-identified medical examiner and laboratory records using the predefined rules below. Assignment was not blinded to the reported gene, variant, or laboratory class. Disagreements were resolved by consensus discussion among the authors; independent dual rating was not performed, and inter-rater reliability was not assessed. The four labels remain author-defined interpretive labels for this series, not a validated clinical score or decision algorithm. For each primary LDS-spectrum variant, phenotype–variant concordance with autopsy (±premortem) phenotype was graded as follows. Strong concordance: autopsy (±premortem) findings highly specific for LDS/FTAAD aortopathy (here, fatal ascending aortic tear, dissection, or rupture, with or without syndromic features), with the variant plausibly related to that mechanism of death. Limited concordance: a primary LDS-spectrum allele is present, but the phenotype is nonspecific for aortopathy, and the certified mechanism is not a fully explanatory non-aortopathy alternative that excludes LDS-typical arterial catastrophe. Discordant: the certified cause of death is a non-aortic mechanism (pulmonary embolism, ethanol-related death, or infant sudden death without LDS-typical arterial catastrophe) without LDS-typical arterial catastrophe, and/or a competing gene/phenotype explanation dominates; this rule is independent of co-reported *VWF* or other hemostasis-gene findings. Not assessable: insufficient autopsy/phenotype data to judge concordance. Minimum data to avoid not assessable were (i) a medical examiner cause of death or equivalent stated mechanism and (ii) documented presence or explicit absence of a major cardiovascular autopsy finding (high-specificity thoracic aortopathy—aneurysm, dissection, or rupture—or an alternate certified cardiovascular/toxicologic mechanism). Incomplete GeneReviews-style feature capture (Supplementary Table S5) does not by itself trigger not assessable. Death itself was not used as circular evidence of pathogenicity. Grades summarize phenotype–variant concordance for LDS-spectrum interpretation and do not equate laboratory-variant detection with a clinical LDS diagnosis. For each case, Results also record three linked interpretive conclusions: (a) variant pathogenicity as reported by the laboratory (without independent ACMG reclassification), (b) phenotype compatibility with the gene-associated LDS/FTAAD-spectrum disorder (yes/limited/no), and (c) contribution of the primary LDS-spectrum variant to the certified mechanism of death (plausible/not established/unlikely), mapped consistently to the concordance grade (strong → yes/plausible; limited → limited/not established; discordant → no/unlikely).

For reporting of additional findings, incidental (or secondary) findings are reportable variants that may be related to an autopsy investigation but do not match the phenotype or circumstances of death. Uncertain denotes additional or CNV findings for which autopsy fit could not be judged confidently from available records. Role labels distinguish the primary LDS/FTAAD-spectrum allele used for ascertainment and concordance grading from additional reportable variants and CNVs on the same molecular report.

### 2.6. In Silico Variant Annotation

Primary LDS-spectrum variants were annotated with population, evolutionary, functional, and structural predictors for supplementary molecular context; these analyses were secondary to phenotype–variant concordance grading. Population annotations included gnomAD allele frequencies and ClinVar clinical significance when available [3,15]. Functional predictors emphasized CADD and AlphaMissense pathogenicity scores [16,17,18]. For expanded variant inventories, CADD and AlphaMissense values were retrieved via the Ensembl Variant Effect Predictor (VEP) REST API (/vep/human/hgvs) with CADD and AlphaMissense plugins enabled [19]; scores were extracted from transcript consequences matching the reported gene and were never invented when VEP fields were absent. Structural stability for primary LDS-spectrum variants was estimated with DynaMut [20] as the predicted change in free energy/stability (ΔΔG, kcal·mol^−1^), with negative values interpreted as destabilizing. CADD, AlphaMissense, and DynaMut ΔΔG are in silico predictions used as supplementary computational context only. Predictor concordance quadrants used thresholds of CADD ≥ 20 and AlphaMissense ≥ 0.564, consistent with calibrated computational evidence frameworks [4]. Predefined score sources, thresholds, and figure/table legends are provided with the corresponding Supplementary Materials. Supplementary Figure S1 reports CADD × AlphaMissense quadrants as computational context and should not be conflated with autopsy concordance grades.

### 2.7. Evidence Triangulation

As supplementary context only, evidence triangulation integrated in silico predictors (CADD/AlphaMissense), ClinVar bucket, DynaMut ΔΔG structural support, and the phenotype–variant concordance grade [3,4,16,17,18,20]. Triangulation heatmaps (Supplementary Table S3) are presented as supplementary molecular context demoted relative to autopsy-led interpretation and do not override phenotype–variant concordance grades.

## 3. Results

### 3.1. Case Ascertainment and Molecular Autopsy Findings

We report eight Florida medical examiner decedents with laboratory-reported heterozygous missense variants in LDS/FTAAD-spectrum genes (Table 1). Cases are ordered by gene/pathway: one TGFBR1 (LDS1), three TGFBR2 (LDS2), three TGFB2 (LDS4), and one SMAD3 (LDS3). Decedents ranged in age from 8 months to 46 years (four males, four females) across Florida medical examiner jurisdictions. Postmortem laboratory classifications were pathogenic in one case (*TGFBR1* p.Arg487Gln, Case 1), likely pathogenic in one case (*TGFBR2* p.Glu428Asp, Case 3), and variants of uncertain significance (VUS) in six cases. Of the eight primary alleles, Case 1 *TGFBR1* p.Arg487Gln is laboratory pathogenic and ClinVar Pathogenic; Case 3 *TGFBR2* p.Glu428Asp is laboratory-likely pathogenic and was not in ClinVar in this series; the remaining six primary alleles are laboratory VUS (ClinVar VUS or not in ClinVar as recorded in Supplementary Table S3). Disease-causing status in the clinical-classification sense is restricted to laboratory-pathogenic or likely-pathogenic alleles (Cases 1 and 3); even then, a pathogenic/likely-pathogenic call is not standalone proof that the variant caused death without strong autopsy concordance. VUS are not claimed to be disease-causing. Parental samples were not available for segregation analysis, and family history was not assessable in all eight cases. Premortem features used here are only those already documented (Case 1 migraines; Case 3 marfanoid habitus and collapse during swim team practice; Case 4 asthma and anxiety; Case 5 depression and attention-deficit/hyperactivity disorder; and Case 8 ethanolism). Phenotype–variant concordance was graded case-by-case as strong, limited, discordant, or not assessable; two cases showed strong concordance (Cases 1 and 3), two limited concordance (Cases 4 and 5), and four discordant presentations (Cases 2 and 6–8). All eight cases met the minimum data rule for avoiding not assessable (medical examiner cause of death or equivalent plus documented presence or explicit absence of a major cardiovascular autopsy finding).

Case 1 (8-year-old male). Autopsy demonstrated a posterior-wall ascending aortic transmural tear measuring 4 cm with dissection extending to the arch and 450 mL of liquid and clotted blood in the pericardial sac, together with mild pectus excavatum, sagittal craniosynostosis, mild ventricular dilation, and a microscopically patent ductus arteriosus with intimal calcifications. Premortem history was limited to migraines; he was found unresponsive in bed. These elements should be distinguished: (1) the autopsy mechanism (ruptured ascending aorta with hemopericardium); (2) syndromic skeletal/craniofacial features on autopsy; (3) laboratory pathogenic *TGFBR1* c.1460G>A (p.Arg487Gln); and (4) the medical examiner’s certified wording of ruptured aortic aneurysm due to Loeys–Dietz syndrome. Item (4) is the pathologist’s synthesis of (1)–(3), not a separate premortem clinical LDS diagnosis. The authors’ concordance grade is an independent interpretive label and is not validated by the certificate, even when the grade and the certificate agree. In this LDS type 1/*TGFBR1* gene category, the primary allele was *TGFBR1* c.1460G>A (p.Arg487Gln), laboratory-classified as pathogenic, with no independent ACMG reclassification performed here. Phenotype–variant concordance: strong.

(a) Variant pathogenicity: Laboratory pathogenic (*TGFBR1* p.Arg487Gln); no independent ACMG reclassification performed here.

(b) Phenotype compatibility with gene-associated disorder: Yes—fatal ascending aortic catastrophe with syndromic skeletal/craniofacial features fits LDS1/*TGFBR1*.

(c) Contribution to death: Plausible—concordance grade strong; variant-related attribution to ruptured ascending aortopathy is supported by the autopsy phenotype together with the laboratory pathogenic *TGFBR1* result, not by treating the certificate wording as an independent premortem clinical LDS diagnosis.

Case 2 (36-year-old male). Autopsy identified bilateral pulmonary emboli and lower-extremity deep vein thrombosis. No aortic dissection, aneurysm, or syndromic connective tissue features were documented on the available report. In the LDS type 2/*TGFBR2* gene category, the primary allele was *TGFBR2* c.1663G>A (p.Glu555Lys), laboratory-classified as VUS. The requisition indicated a WES predefined gene set product. Under the explicit rule that a certified non-aortic cause of death (here, pulmonary thromboembolism) without LDS-typical arterial catastrophe is discordant—the same rule as Case 6 saddle pulmonary embolism—phenotype–variant concordance is discordant, independent of co-reported *VWF* findings.

(a) Variant pathogenicity: Laboratory VUS (*TGFBR2* p.Glu555Lys).

(b) Phenotype compatibility with gene-associated disorder: No—pulmonary embolism and deep vein thrombosis without documented aortopathy are not compatible with LDS2 aortopathy as the cause of death.

(c) Contribution to death: Unlikely contribution of *TGFBR2* to death—concordance grade discordant; the primary allele does not explain the certified PE/DVT mechanism.

Case 3 (16-year-old male). Autopsy demonstrated massive hemopericardium from ruptured dissection of the ascending aorta—a high-specificity autopsy phenotype for hereditary aortopathy in the LDS/FTAAD spectrum. Premortem marfanoid habitus was documented, and he collapsed during swim team practice. In the LDS type 2/*TGFBR2* gene category, the primary allele was *TGFBR2* c.1284A>T (p.Glu428Asp), laboratory-classified as likely pathogenic, with laboratory interpretation language consistent with Loeys–Dietz syndrome on the source report. Together with laboratory-likely-pathogenic classification in *TGFBR2*, this case represents the strongest concordant case in the series. Phenotype–variant concordance: strong.

(a) Variant pathogenicity: Laboratory-likely pathogenic (*TGFBR2* p.Glu428Asp).

(b) Phenotype compatibility with gene-associated disorder: Yes—ruptured ascending dissection with marfanoid habitus fits LDS2/*TGFBR2*.

(c) Contribution to death: Plausible—concordance grade strong; supports consideration of family cardiovascular genetics follow-up pending clinical confirmation, distinct from a formal premortem clinical LDS diagnosis outside laboratory report language.

Case 4 (34-year-old female). She was found unresponsive with pulseless electrical activity; available examination did not document hypertelorism, bifid uvula, or other classic craniofacial LDS stigmata. No aortic dissection or aneurysm was stated; pulmonary microthrombi and cerebral edema were noted in source materials, and cause of death was indeterminate in the legacy clinical summary. Premortem history included asthma and anxiety. In the LDS type 2/*TGFBR2* gene category, the primary allele was *TGFBR2* c.652A>G (p.Ile218Val), laboratory-classified as VUS. Assay product was recorded as WES (phenotype-based) on the laboratory information system line. Phenotype–variant concordance: limited (nonspecific phenotype without a certified alternative that excludes aortopathy).

(a) Variant pathogenicity: Laboratory VUS (*TGFBR2* p.Ile218Val).

(b) Phenotype compatibility with gene-associated disorder: Limited—sudden death without documented aortopathy is nonspecific for LDS2.

(c) Contribution to death: Not established—concordance grade limited.

Case 5 (32-year-old female). Autopsy showed mild cardiomegaly without documented aortic or arterial LDS-related pathology. Premortem history included depression and attention-deficit/hyperactivity disorder. In the LDS type 4/*TGFB2* gene category, the primary allele was *TGFB2* c.5A>G (p.His2Arg), laboratory-classified as VUS. Phenotype–variant concordance: limited.

(a) Variant pathogenicity: Laboratory VUS (*TGFB2* p.His2Arg).

(b) Phenotype compatibility with gene-associated disorder: Limited—mild cardiomegaly alone is nonspecific for LDS4.

(c) Contribution to death: Not established—concordance grade limited.

Case 6 (46-year-old male). Autopsy identified saddle pulmonary embolism with clinical suspicion of thrombophilia. No aortic LDS-spectrum vasculopathy was documented. In the LDS type 4/*TGFB2* gene category, the primary allele was *TGFB2* c.645C>G (p.Asp215Glu), laboratory-classified as VUS. The laboratory indication documented a Focus product with a thrombophilia-focused panel context. Phenotype–variant concordance: discordant (thrombotic cause of death pointing away from LDS aortopathy).

(a) Variant pathogenicity: Laboratory VUS (*TGFB2* p.Asp215Glu).

(b) Phenotype compatibility with gene-associated disorder: No—saddle embolism/thrombophilia presentation does not match LDS4 aortopathy.

(c) Contribution to death: Unlikely—concordance grade discordant.

Case 7 (8-month-old female). Death occurred in an infant sudden-death context without documented aortic LDS-spectrum vasculopathy on available records. In the LDS type 4/*TGFB2* gene category, the primary allele was *TGFB2* c.624T>A (p.His208Gln), laboratory-classified as VUS. Phenotype–variant concordance for the *TGFB2* primary LDS-spectrum variant: discordant (*TGFB2* not treated as causal for the certified infant presentation). Separately, the same molecular report included heterozygous *MYOM1* c.3199C>T (p.Arg1067Trp), laboratory VUS, with report language associating *MYOM1* variation with hypertrophic cardiomyopathy; laboratory evidence cited ClinVar uncertain significance and a low gnomAD GroupMax frequency (0.001% in South Asian). There is insufficient autopsy phenotype evidence to attribute cardiomyopathy to the *MYOM1* allele. Thus, (i) primary LDS-spectrum *TGFB2* is an ascertainment finding, not a cause-of-death attribution; (ii) *MYOM1* is a co-reported cardiomyopathy-gene finding; and (iii) death is not attributed to either allele.

(a) Variant pathogenicity: Laboratory VUS (*TGFB2* p.His208Gln).

(b) Phenotype compatibility with gene-associated disorder: No—infant sudden death without LDS aortopathy does not match LDS4.

(c) Contribution to death: Unlikely—concordance grade discordant for the primary *TGFB2* allele.

Case 8 (21-year-old female). Medical examiner review documented a normal-caliber aorta without aneurysms and hepatic macro- and microvesicular steatosis with mild sinusoidal congestion, and cause of death was certified as ethanol withdrawal due to ethanolism, with an explicit statement of no evidence of Loeys–Dietz syndrome. In the LDS type 3/*SMAD3* gene category, the primary allele was *SMAD3* c.458T>A (p.Leu153Gln), laboratory-classified as VUS. Phenotype–variant concordance: discordant.

(a) Variant pathogenicity: Laboratory VUS (*SMAD3* p.Leu153Gln).

(b) Phenotype compatibility with gene-associated disorder: No—normal aorta and ethanol-related cause of death are incompatible with LDS3 aortopathy.

(c) Contribution to death: Unlikely—concordance grade discordant.

Strong concordance with LDS-compatible presentations was confined to the two aortic-catastrophe decedents. Case 1 carried *TGFBR1* p.Arg487Gln with ruptured ascending aortic dissection, hemopericardium, and syndromic skeletal/craniofacial features; the medical examiner certified ruptured aortic aneurysm due to Loeys–Dietz syndrome as a synthesis of autopsy phenotype plus the laboratory pathogenic *TGFBR1* result, not as a premortem clinical LDS diagnosis. Pathogenic missense variation in the *TGFBR1* serine/threonine kinase domain is a well-established mechanism of LDS type 1 aortopathy [9,10,11,21]. Case 3 remains the demonstration case for classic heritable aortopathy: premortem marfanoid habitus, collapse during exertion, and ruptured ascending dissection with massive hemopericardium, together with a likely-pathogenic *TGFBR2* kinase-domain variant (p.Glu428Asp) [9,11,21,22]. In both cases, autopsy phenotype was highly specific for LDS/FTAAD-spectrum aortopathy, supporting plausible variant-related attribution to the cause of death. Even for these pathogenic/likely-pathogenic alleles, concordance is not biochemical proof.

### 3.2. Autopsy-Led Concordance Grading

**Figure 1:**
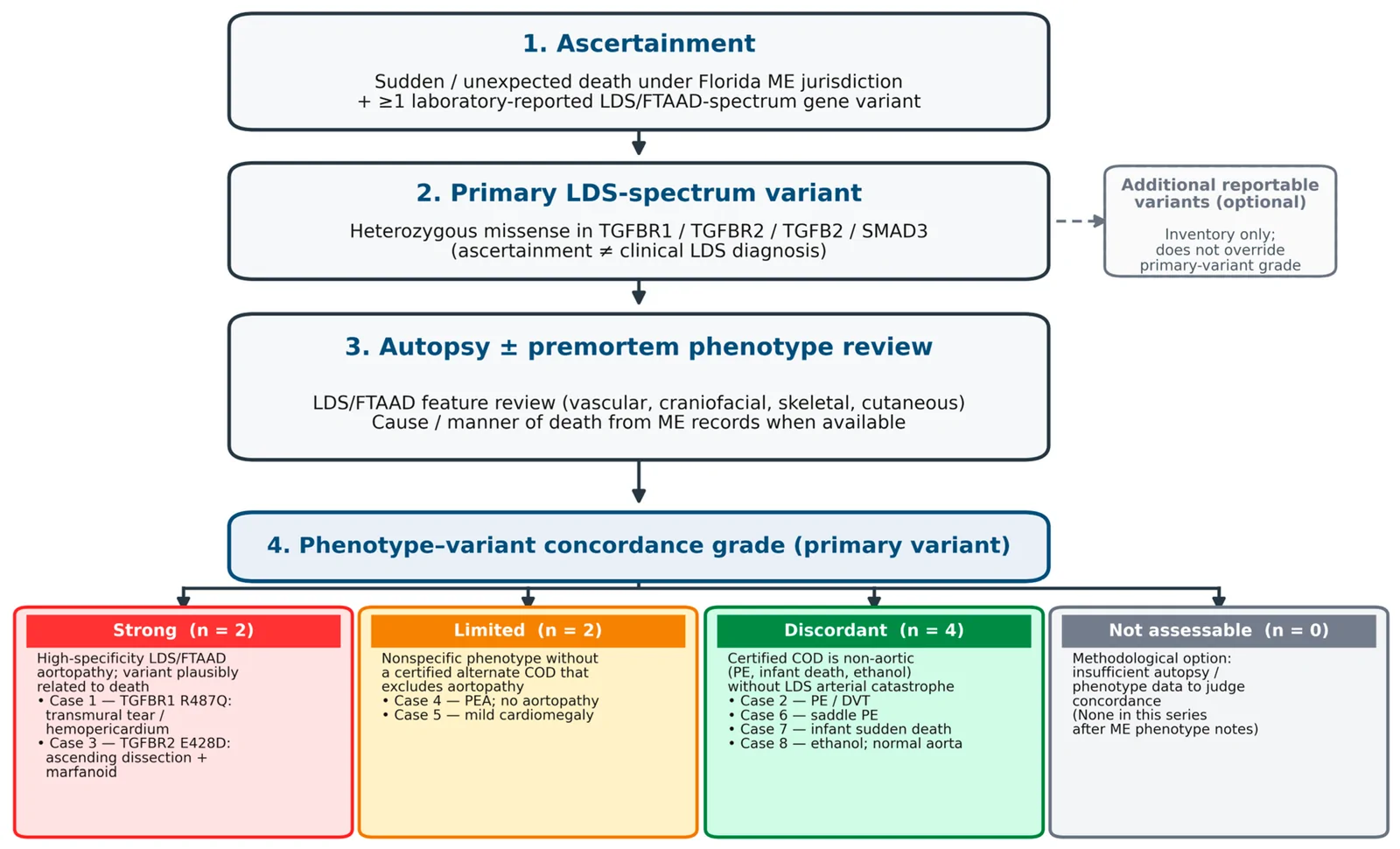
Proposed interpretive framework for autopsy-led interpretation of postmortem LDS-spectrum variants. Pipeline from ascertainment and primary LDS/FTAAD-spectrum variant through autopsy phenotype review to concordance grades (strong/limited/discordant/not assessable). Optional additional reportable variants do not override the primary-variant grade. Solid arrows show the main workflow; the dashed arrow indicates optional additional reportable variants. In this series: strong *n* = 2 (Cases 1 and 3); limited *n* = 2 (Cases 4 and 5); discordant *n* = 4 (Cases 2, 6, 7, and 8); not assessable *n* = 0. This figure is a proposed interpretive framework used to report this variant-positive series; it is not a validated clinical decision algorithm or a mutation-causality or pathway-flux diagram, and it has not been evaluated for inter-rater reliability or prospective performance. With *n* = 8, allele-to-death inference remains illustrative.

Figure 1 summarizes the proposed interpretive framework for the eight Florida medical examiner decedents (not a validated decision algorithm). In this series, grades were mapped as strong in two cases (Cases 1 and 3), limited in two (Cases 4 and 5), and discordant in four (Cases 2 and 6–8); no case was graded not assessable. All eight cases met the minimum data rule (stated mechanism plus assessable presence or absence of high-specificity aortopathy or an alternate mechanism). GeneReviews-style feature completeness (Supplementary Table S5) was often “not documented” and does not by itself trigger not assessable. Interpretive constraints are greatest for Cases 4–8: phenotyping is incomplete (no premortem aortic imaging; limited craniofacial/skeletal/cutaneous capture), so grades rest on absence of high-specificity aortopathy plus the certified mechanism and are less robust than Cases 1 and 3. Cases 4 and 5 remain limited; Cases 6–8 (and Case 2 after equalization of PE-without-aortopathy) remain discordant rather than not assessable because the minimum data floor was met.

Table 2 lists the eight primary LDS/FTAAD-spectrum missense alleles together with, at most, two competing or phenotype-adjacent additional findings discussed in the text: the Case 2 *VWF* copy-number variant and the Case 7 *MYOM1* VUS. The complete laboratory report inventory (*n* = 40 rows; 8 primary, 31 additional, and 1 CNV) is in Supplementary Table S7. Role distinguishes primary from additional and CNV tiers. Columns add a short primary autopsy finding and, for primary rows, the phenotype–variant concordance grade or, for additional/CNV rows, the secondary incidental note (Supplementary Table S4). Additional findings are contextualized relative to autopsy phenotype in Supplementary Table S4; broad LDS feature scoring is in Supplementary Table S5; per-case molecular testing, interpretation scope, coverage, and CNV documentation are in Supplementary Table S6.

### 3.3. Molecular Contextual Evidence

In silico annotations were reviewed as secondary molecular context only and are not biochemical proof. On the joint CADD × AlphaMissense landscape (Supplementary Figure S1; thresholds CADD ≥ 20 and AlphaMissense ≥ 0.564), the eight primary missense alleles occupied distinct predictor-concordance quadrants that only partly aligned with phenotype–variant concordance. Those quadrants must not be read as autopsy grades. Population and evolutionary annotations are summarized in Supplementary Table S1; functional, sequence-context, and structural scores are in Supplementary Table S2. Supplementary Table S3 integrates predictor concordance, ClinVar, ΔΔG, and the phenotype–variant concordance grade on a single triangulation panel.

The two strong-concordance aortic cases occupied the concordant-pathogenic predictor quadrant. Case 1 (*TGFBR1* p.Arg487Gln) had CADD 31 and AlphaMissense 0.995, with ClinVar Pathogenic classification and ΔΔG −0.63 kcal·mol^−1^. Case 3 (*TGFBR2* p.Glu428Asp) had CADD 22.5 and AlphaMissense 0.9957, with laboratory-likely-pathogenic classification (not in ClinVar) and ΔΔG −1.04 kcal·mol^−1^. In Supplementary Tables S1–S3, these alleles therefore combine the strongest multi-axis molecular support with the only high-specificity aortic phenotypes in the series.

Limited-concordance Cases 4 and 5 illustrate the more common scenario: a relevant LDS-spectrum gene finding without high-specificity aortopathy and without a certified alternate mechanism that excludes aortopathy. Case 4 (*TGFBR2* p.Ile218Val VUS) presented with indeterminate sudden death without documented dissection or aneurysm; Case 5 (*TGFB2* p.His2Arg VUS) presented with mild cardiomegaly alone. *TGFB2* loss-of-function and related alleles can cause familial thoracic aortopathy/LDS type 4 [13,14,23], but nonspecific sudden death without aortic catastrophe does not upgrade a VUS to cause-of-death attribution [2,7].

By contrast, molecular scores among discordant Cases 2 and 6–8 did not track phenotype–variant concordance in a uniform direction. Case 2 (*TGFBR2* p.Glu555Lys VUS) and Case 6 (*TGFB2* p.Asp215Glu VUS) were certified thromboembolic deaths without documented aortopathy; Case 6 fell in the concordant-benign predictor quadrant (CADD 14.63; AlphaMissense 0.1408). Case 7 (*TGFB2* p.His208Gln) likewise scored as concordant-benign (CADD 0.196; AlphaMissense 0.0766). Case 8 (*SMAD3* p.Leu153Gln) showed CADD–AlphaMissense discordance (CADD 29.5; AlphaMissense 0.39) despite a medical examiner-documented normal aorta and ethanol-related cause of death. Triangulation (Supplementary Table S3), therefore, separates Cases 1 and 3—strong phenotype plus concordant-pathogenic predictors—from Cases 2 and 6–8, in which alternate mechanisms of death dominate irrespective of whether in silico scores are low (Cases 6–7) or partly elevated (Cases 2 and 8). These patterns illustrate that high in silico scores alone do not establish LDS-spectrum attribution when autopsy phenotype is discordant and that low scores among discordant *TGFB2* alleles are consistent with—but secondary to—the autopsy-led grade.

### 3.4. Secondary and Additional Variants

Beyond the eight primary alleles, Supplementary Table S7 inventories all additional reportable findings and the single copy-number variant from the same molecular autopsy reports; Table 2 shows only the primary alleles plus the Case 2 *VWF* CNV and Case 7 *MYOM1* VUS. Case 1 had no additional findings; Cases 2–8 carried one or more additional variants. Supplementary Table S4 scores incidental context of these secondary findings relative to each decedent’s autopsy phenotype.

Among secondary findings, Case 2 was ascertained on the laboratory-reported *TGFBR2* VUS (primary LDS-spectrum allele; discordant concordance; bilateral pulmonary emboli and lower-extremity deep vein thrombosis; Table 2). The same molecular report also listed two *VWF* findings: a heterozygous likely-pathogenic exon deletion and a co-reported *VWF* missense VUS. As heterozygous laboratory-likely-pathogenic *VWF* exon deletion and missense variants were co-reported, but without VWF assays or a documented hematologic diagnosis, this finding is insufficient to establish von Willebrand disease or a defined thrombophilia and is recorded only as a competing hemostasis-gene context, not as a causal explanation of PE/DVT. That autopsy-context note does not replace the primary LDS-spectrum ascertainment call or convert the *TGFBR2* VUS into strong LDS concordance. Across the remaining secondary inventory, most calls were incidental heterozygous carrier findings (including pathogenic or likely-pathogenic alleles in recessive disease genes without a second allele) or otherwise non-explanatory for the certified cause of death, including secondary findings co-reported with strong-concordance Case 3, which do not displace the primary *TGFBR2* aortopathy attribution. Secondary incidental assessments, therefore, contextualize additional/secondary report content; they are not applied to primary LDS-spectrum alleles.

## 4. Discussion

This illustrative, variant-positive Florida medical examiner series comprises eight unrelated decedents with laboratory-reported heterozygous missense variants in LDS/FTAAD-spectrum genes (*TGFBR1*, *TGFBR2*, *TGFB2*, or *SMAD3*). None had a premortem diagnosis of LDS. Laboratory detection of a rare LDS-relevant variant does not establish clinical LDS or cause of death; autopsy (±premortem) phenotype leads, with laboratory class and in silico scores as secondary context only [1,2,8]. Author-assigned concordance grades (not blinded to laboratory class; reconciled by discussion) yielded strong concordance in Cases 1 and 3, limited concordance in Cases 4 and 5, and discordant presentations in Cases 2 and 6–8. This series cannot estimate prevalence, diagnostic yield, or penetrance.

Strong concordance was confined to the two aortic-catastrophe decedents. Case 1 carried *TGFBR1* p.Arg487Gln (laboratory pathogenic; ClinVar Pathogenic) with ruptured ascending aortic dissection, hemopericardium, and syndromic skeletal/craniofacial features [9,11]. The medical examiner certified a ruptured aortic aneurysm due to Loeys–Dietz syndrome; that wording synthesizes autopsy mechanism, syndromic features, and the pathogenic *TGFBR1* result—not a premortem clinical LDS diagnosis—and the authors’ strong grade is independent of the certificate even when they agree. Case 3 had premortem marfanoid habitus, exertional collapse, ruptured ascending dissection with massive hemopericardium, and a likely-pathogenic *TGFBR2* kinase-domain variant (p.Glu428Asp; not in ClinVar in this series) [11,22]. Even pathogenic/likely-pathogenic laboratory labels require strong autopsy concordance for cause-of-death attribution.

Limited-concordance Cases 4 and 5 illustrate an LDS-spectrum gene finding without high-specificity aortopathy or a fully explanatory alternate certified mechanism. Case 4 (*TGFBR2* p.Ile218Val VUS) had indeterminate sudden death with pulseless electrical activity and no documented dissection or aneurysm; Case 5 (*TGFB2* p.His2Arg VUS) had mild cardiomegaly alone. TGFB2 loss of function and related alleles can cause familial thoracic aortopathy/LDS type 4 [13,14,23], but nonspecific sudden death without aortic catastrophe does not upgrade a VUS to cause-of-death attribution. Grades for Cases 4–8 rest on the certified mechanism and documented cardiovascular findings rather than a complete LDS workup and are less robust than Cases 1 and 3.

Discordant Cases 2, 6, 7, and 8 have certified mechanisms that are not LDS-typical arterial catastrophe. Cases 2 and 6 have pulmonary thromboembolism (bilateral PE/DVT and saddle PE, respectively) without documented aortopathy. A heterozygous laboratory-likely-pathogenic *VWF* exon deletion co-reported in Case 2 is recorded only as competing hemostasis-gene context and does not establish von Willebrand disease or a defined thrombophilia. Case 7 occurred in an infant sudden-death setting: (i) the primary LDS-spectrum allele *TGFB2* p.His208Gln (VUS) is not treated as causal; (ii) co-reported *MYOM1* p.Arg1067Trp (VUS; cardiomyopathy-gene context on the laboratory report) is phenotype-adjacent in principle but is not supported by documented hypertrophic cardiomyopathy in this decedent; and (iii) neither allele is established as the mechanism of death. Case 8 carried *SMAD3* p.Leu153Gln (VUS). Pathogenic *SMAD3* variation underlies LDS type 3/aneurysm–osteoarthritis syndrome with thoracic aortic disease [12,24], yet medical examiner review documented a normal-caliber aorta and certified ethanol withdrawal.

Environmental and toxicological contributors could not be systematically assessed because exposure histories and toxicology data were not uniformly available in the de-identified records. Where a clearly documented non-genetic mechanism was present, it was incorporated into the concordance assessment, for example, Case 8′s normal-caliber aorta and certified ethanol withdrawal, supporting discordance between the *SMAD3* VUS and the mechanism of death.

The four genes represented in this series occupy different levels of canonical TGF-β signaling: TGFB2 encodes ligand, TGFBR2 and TGFBR1 encode the type II and type I receptors, and SMAD3 mediates downstream transcriptional signaling. Pathogenic variation in these genes can therefore converge biologically on dysregulated TGF-β signaling and connective tissue/aortic disease [9,10,11,12,13,14]. However, pathway convergence does not establish that every variant identified here is pathogenic or that every death resulted from the same mechanism; six of the eight primary variants were VUS, and most cases lacked LDS-typical aortic pathology.

Molecular annotation (Supplementary Figure S1; Supplementary Tables S1–S3) was retained as secondary context. Joint CADD × AlphaMissense positioning and ClinVar buckets [3,16,18] aligned with strong aortic cases but did not rescue limited or discordant grades when autopsy argued otherwise. DynaMut ΔΔG estimates are supplementary stability predictions, not experimental assays [20]. Computational tools support PP3/BP4-style evidence [4] but do not replace autopsy-led concordance grading [2].

Secondary variants are inventoried in Supplementary Table S7 and contextualized in Supplementary Table S4 without overriding primary concordance grades; most were incidental carrier calls or non-explanatory for the certified cause of death [1,2,7].

For medical examiner and molecular autopsy workflows, rare *TGFBR1*, *TGFBR2*, *TGFB2*, or *SMAD3* variants should be distinguished from clinical LDS diagnosis, actionable classification, and cause-of-death attribution [2,8,11]. Autopsy-led concordance grading offers a practical scaffold: escalate family cardiovascular genetics when autopsy shows high-specificity aortopathy with a concordant allele (Cases 1 and 3) and avoid overcalling incidental findings when phenotype is limited or discordant.

Several limitations follow from the design. This variant-positive convenience series (*n* = 8) is not a consecutive sudden-death cohort. Although all eight cases underwent WES, interpretation/reporting scope and recovered coverage varied (Supplementary Table S6); complete gene lists were not recovered for several cases and were unavailable. Family history and parental samples were unavailable (PP1/segregation not applied). Without family testing, attribution remains incomplete even for Cases 1 and 3 and for the remaining six cases. In silico models do not replace functional validation or clinical LDS criteria [11]. Concordance grades were assigned unblinded using predefined rules, with disagreements handled by discussion; inter-rater reliability was not quantified, and the labels are not a validated clinical score. External validation requires larger series with structured phenotype capture [1,2].

## 5. Conclusions

Only two of the eight cases (Case 1, *TGFBR1* p.Arg487Gln, fatal ascending aortic catastrophe; Case 3, *TGFBR2* p.Glu428Asp, ruptured ascending dissection) showed strong autopsy-led support for LDS-spectrum attribution to the mechanism of death; two cases were limited (Cases 4 and 5), and four were discordant (Cases 2 and 6–8), including *SMAD3* (Case 8) with ethanol-related death and a normal aorta. Autopsy-led concordance grading, applied without using death as evidence of pathogenicity and without equating laboratory detection with clinical LDS diagnosis, may help medical examiner and molecular autopsy workflows identify heritable risk. Rare *TGFBR1*, *TGFBR2*, *TGFB2*, or *SMAD3* variants should not be conflated with an LDS diagnosis or with the cause of death without concordant autopsy evidence. Because this was an illustrative, variant-positive Florida medical examiner series rather than a consecutive sudden-death cohort, it cannot be used to estimate prevalence, diagnostic yield, penetrance, or incidental-finding frequency.

## Figures and Tables

**Table 1:** Clinical and primary-variant summary of eight Florida medical examiner decedents with postmortem LDS/FTAAD-spectrum gene findings (gene/pathway order).

Case	Age/Sex	Gene	Variant	Domain	Lab Class
1	8 y/M	*TGFBR1*	c.1460G>A (p.Arg487Gln)	Serine/threonine protein kinase domain	Pathogenic
2	36 y/M	*TGFBR2*	c.1663G>A (p.Glu555Lys)	C-terminal tail/distal kinase domain boundary	VUS
3	16 y/M	*TGFBR2*	c.1284A>T (p.Glu428Asp)	Serine/threonine kinase domain	Likely pathogenic
4	34 y/F	*TGFBR2*	c.652A>G (p.Ile218Val)	Juxtamembrane cytoplasmic region (pre-kinase domain)	VUS
5	32 y/F	*TGFB2*	c.5A>G (p.His2Arg)	Signal peptide/N-terminal propeptide	VUS
6	46 y/M	*TGFB2*	c.645C>G (p.Asp215Glu)	LAP/propeptide domain	VUS
7	8 m/F	*TGFB2*	c.624T>A (p.His208Gln)	LAP/propeptide domain	VUS
8	21 y/F	*SMAD3*	c.458T>A (p.Leu153Gln)	MH1–MH2 linker	VUS

Abbreviations: M, male; F, female; y, years; m, months; Lab Class, postmortem laboratory classification; VUS, variant of uncertain significance; LAP, latency-associated peptide/propeptide domain (TGFB2). Domain annotations follow UniProt. Geography below the Florida state level is omitted for privacy. No patient names. Table 1 summarizes ascertainment alleles and laboratory class. Predicted stability (DynaMut ΔΔG) and pathogenicity scores (CADD, AlphaMissense) are in Supplementary Tables S1–S3 and are computational only.

**Table 2:** Primary LDS/FTAAD-spectrum alleles and selected competing findings (Case 2 *VWF* CNV; Case 7 *MYOM1*) for eight Florida medical examiner decedents, with primary autopsy finding and concordance/incidental notes. The full 40-row laboratory inventory is in Supplementary Table S7.

Case	Primary Autopsy Finding	Gene	HGVS	Zyg.	Lab Class	Role	Concordance/Incidental
1	Ruptured ascending aortic tear/dissection; hemopericardium	*TGFBR1*	c.1460G>A (p.Arg487Gln)	Het	P	primary	strong
2	Bilateral PE + LE DVT	*TGFBR2*	c.1663G>A (p.Glu555Lys)	Het	VUS	primary	discordant
	Bilateral PE + LE DVT	*VWF*	12:g.(?_6127521)_(6134874_?)del	Het	LP	CNV	competing *VWF* CNV; VWD/thrombophilia not established
3	Ruptured ascending aortic dissection; hemopericardium	*TGFBR2*	c.1284A>T (p.Glu428Asp)	Het	LP	primary	strong
4	Sudden death (PEA); COD indeterminate	*TGFBR2*	c.652A>G (p.Ile218Val)	Het	VUS	primary	limited
5	Mild cardiomegaly; sudden demise	*TGFB2*	c.5A>G (p.His2Arg)	Het	VUS	primary	limited
6	Saddle pulmonary embolism	*TGFB2*	c.645C>G (p.Asp215Glu)	Het	VUS	primary	discordant
7	Sudden infant death	*TGFB2*	c.624T>A (p.His208Gln)	Het	VUS	primary	discordant
	Sudden infant death	*MYOM1*	c.3199C>T (p.Arg1067Trp)	Het	VUS	additional	uncertain (CMP gene; death not attributed)
8	Ethanol withdrawal; normal-caliber aorta	*SMAD3*	c.458T>A (p.Leu153Gln)	Het	VUS	primary	discordant

Case = manuscript case number (1–8), identical to Table 1. Primary autopsy finding = short autopsy phrase (repeated within each case block). Extraction C-labels (C1–C8) are for laboratory-source traceability only (case_order_map.csv); C5 = *SMAD3* = Case 8 (not Case 5). Role: primary = LDS/FTAAD-spectrum ascertainment allele used for concordance grading; additional = secondary reported variant on the same report; CNV = copy-number variant. Concordance/incidental: primary rows report phenotype–variant concordance grade (strong/limited/discordant); additional/CNV rows report incidental or autopsy-context notes (Supplementary Table S4). Case 2 *VWF* CNV is recorded as competing hemostasis-gene context only; von Willebrand disease and thrombophilia are not established from these records. Incidental (or secondary) findings are reportable variants that may be related to an autopsy investigation but do not match the phenotype or circumstances of death; uncertain denotes findings for which an autopsy fit could not be judged confidently from available records. ClinVar buckets and in silico flags (CADD × AlphaMissense quadrant; DynaMut ΔΔG) for primary alleles are in Supplementary Table S3 and are computational predictions. Disease-causing in the clinical-classification sense is restricted to laboratory-pathogenic or likely-pathogenic alleles (Cases 1 and 3); even those two calls are not used as standalone proof that the variant caused death without strong autopsy concordance. VUS are not described as disease-causing. Interpretation of additional or negative gene findings is limited to genes included in the documented analysis/reporting scope (Supplementary Table S6). Abbreviations: Het, heterozygous; P, pathogenic; LP, likely pathogenic; VUS, variant of uncertain significance. No patient names.

## Data Availability

The data supporting the findings of this study are not publicly available due to ethical and privacy restrictions related to postmortem genomic information. The raw genomic data are not available for public sharing, as whole-exome sequencing was performed by an external clinical laboratory, and only final reports were provided to the authors. No raw sequencing files (e.g., FASTQ, BAM, or VCF) are available.

## Supplementary Materials

The following supporting information can be downloaded at: https://www.mdpi.com/article/10.3390/genes17091040/s1: Supplementary Figure S1: CADD × AlphaMissense in silico landscape (computational quadrants, not autopsy grades); Supplementary Table S1: population and evolutionary annotations; Supplementary Table S2: functional, sequence-context, and structural in silico scores (DynaMut ΔΔG); Supplementary Table S3: evidence triangulation with phenotype–variant concordance grades (Case 2 discordant; 2 strong/2 limited/4 discordant); Supplementary Table S4: autopsy-context fit of secondary variants (*VWF* recorded as competing hemostasis-gene context only); Supplementary Table S5: broad LDS/FTAAD feature review; Supplementary Table S6: per-case molecular testing, interpretation scope, coverage, and CNV documentation; Supplementary Table S7: complete 40-row laboratory-report variant inventory.
