# Supplementary material for: Illustrative Variant-Positive Molecular Autopsy Series: Autopsy-Led Concordance of Loeys–Dietz Spectrum Findings in Eight Florida Sudden Deaths
Source: Genes (Basel). 2026 Aug 29;17(9):1040. doi: 10.3390/genes17091040 (PMC13606872; doi:10.3390/genes17091040)
Supplement: Supplementary file 1 [file genes-17-01040-s001.zip › genes-4503526-supplementary.pdf]

Supplementary Materials

Illustrative Variant-Positive Molecular Autopsy Series: Autopsy-Led Concordance of Loeys–Dietz Spectrum Findings in Eight Florida Sudden Deaths

Overview

This supplement collects molecular and secondary-variant materials demoted from the main text for an illustrative variant-positive molecular autopsy series of eight Florida medical examiner decedents with laboratory-reported Loeys–Dietz syndrome (LDS) / familial thoracic aortic aneurysm and dissection (FTAAD)–spectrum gene findings. The main text foregrounds autopsy (± premortem) phenotype–variant concordance grading (strong n = 2, Cases 1 and 3; limited n = 2, Cases 4 and 5; discordant n = 4, Cases 2, 6, 7, and 8; not assessable n = 0). Case 2 (PE/DVT without aortopathy) is graded discordant under the same rule as Case 6 (saddle PE). In silico panels (Supplementary Figure S1; Tables S1–S3) are computational context and are not autopsy grades. Supplementary Table S6 summarizes per-case molecular testing, interpretation scope, coverage, and CNV documentation rebuilt from laboratory reports (all eight cases WES; heterogeneity is downstream interpretation/reporting). Supplementary Table S7 holds the full 40-row laboratory inventory previously in main-text Table 2.

Supplementary Figure S1. CADD × AlphaMissense pathogenicity landscape for eight Florida primary missense variants.

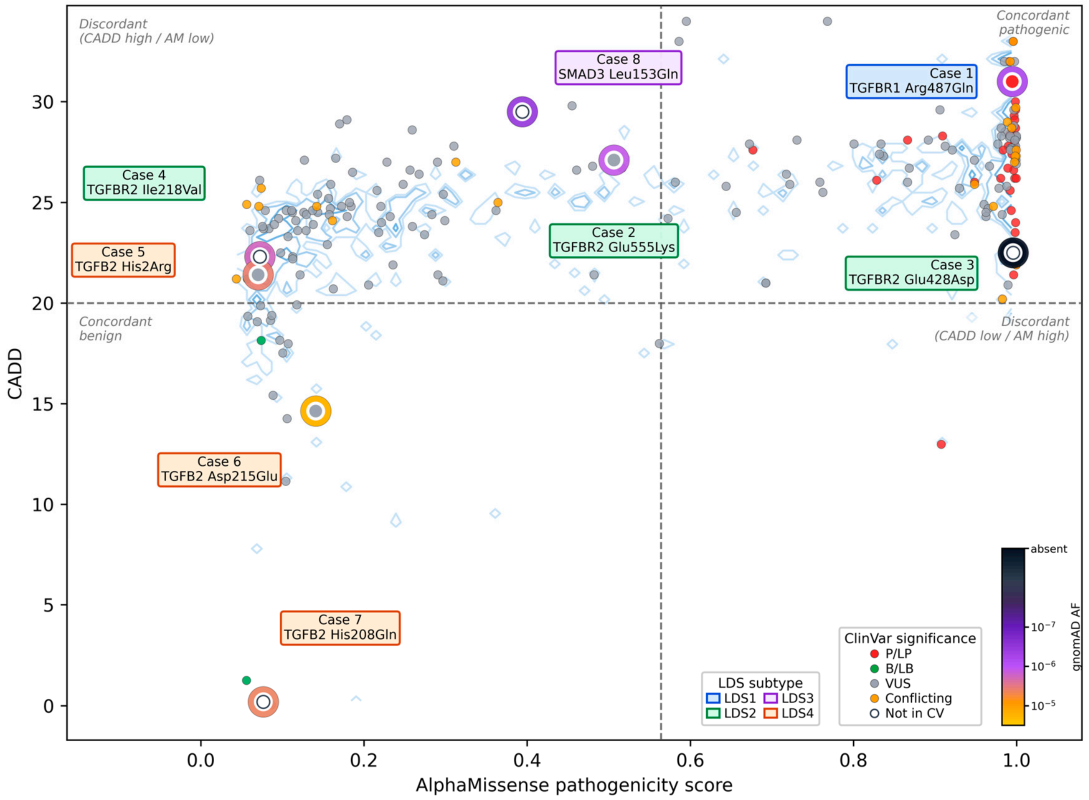

Background cloud: all local GRCh38 CADD v1.7 CodingTranscript NON_SYNONYMOUS missense SNVs in ±50 bp windows around the primary alleles for TGFBR1, TGFBR2, TGFB2, and SMAD3 that successfully joined to AlphaMissense (n = 1,302; TGFBR2 n = 639, TGFB2 n = 225, SMAD3 n = 220, TGFBR1 n = 218). Axes: AlphaMissense pathogenicity score (x) versus CADD (y). Dashed lines mark reference thresholds (AlphaMissense = 0.564; CADD = 20) and define four predictor-concordance quadrants. Two-dimensional isodensity contours mark the 50th, 75th, and 90th percentiles of the background distribution. Background points are coloured by collapsed ClinVar bucket (Pathogenic/Likely pathogenic, Benign/Likely benign, VUS, Conflicting, or Not in ClinVar). The eight primary LDS-spectrum variants (Cases 1–8) are overlaid as corona markers (outer ring = gnomAD v4.1.1 allele-frequency rarity on a $-\log_{10}$(AF) scale; centre fill = ClinVar bucket) with LDS-subtype–bordered callouts (LDS1 blue, LDS2 green, LDS3 purple, LDS4 orange). Case 7 (TGFB2 p.His208Gln) lies outside the downloaded TGFB2 CADD windows and uses documented fallback scores (CADD 0.196; AlphaMissense 0.0766). Numerical annotations are expanded in Supplementary Tables S1 and S2. Predictor positions provide molecular context only. Note: CADD × AlphaMissense quadrants are in silico computational categories and must not be conflated with autopsy-led concordance grades (strong / limited / discordant / not assessable).

Supplementary Table S1. Population and evolutionary *in silico* annotations for the eight primary variants.

Variant information			Population Evidence			Evolutionary Evidence			
Case ID	Gene	Variant	gnomAD AF	ClinVar class	Franklin ACMG	PhyloP100	Sequence Entropy	GERP++	gnomAD Z
Case 1	*TGFBR1*	R487Q	6.20E-07	Pathogenic	Pathogenic	10.00	0.03	5.66	3.99
Case 2	*TGFBR2*	E555K	1.24E-06	VUS	VUS	10.00	0.52	5.14	2.24
Case 3	*TGFBR2*	E428D	N/A	Not in ClinVar	Likely pathogenic	0.57	0.10	3.51	2.24
Case 4	*TGFBR2*	I218V	1.86E-06	Not in ClinVar	VUS	5.71	0.43	5.43	2.24
Case 5	*TGFB2*	H2R	3.79E-06	VUS	VUS	6.78	1.99	4.46	2.13
Case 6	*TGFB2*	D215E	1.55E-05	VUS	VUS	0.19	1.94	3.07	2.13
Case 7	*TGFB2*	H208Q	4.34E-06	Not in ClinVar	VUS	-4.79	2.22	-11.80	2.13
Case 8	*SMAD3*	L153Q	1.24E-06	VUS	VUS	7.88	1.98	5.14	2.24

Supplementary Table S1 summarizes population and evolutionary in silico evidence for the eight Florida primary missense variants (Cases 1–8; gene/pathway order TGFBR1 → TGFBR2 → TGFB2 → SMAD3). Columns report case identifier, gene, protein change, gnomAD allele frequency, ClinVar clinical significance, Franklin ACMG classification, and evolutionary constraint metrics (PhyloP100 vertebrate score, sequence entropy, GERP++ rejection score, and gnomAD gene-constraint Z-score). Numerical values are displayed to two decimal places where applicable; very rare allele frequencies are shown in scientific notation. Cell background colours use a traffic-light scheme: ClinVar/Franklin Pathogenic or Likely pathogenic → pink/red; VUS → grey; Not in ClinVar / N/A → unshaded. PhyloP100: >7 pink, 2–7 yellow, <2 green. Sequence entropy: <0.30 pink, 0.30–1.00 yellow, >1.00 green. GERP++: >4 pink, 2–4

yellow, <2 green. gnomAD Z: >3 pink, 2–3 yellow, <2 green. Missing/N/A numeric cells are grey. Values for Cases 2–7 remapped from the audited legacy Florida evidence table; Cases 1 and 8 filled from the master score paste (mapped by gene/variant, not by legacy paste case ID).

Supplementary Table S2. Functional, sequence-context, and structural *in silico* evidence for the eight primary variants.

Variant information			Functional Evidence				Sequence context			Structural stability
**Case ID**	**Gene**	**Variant**	**CADD**	**Alpha Missense**	**REVEL**	**SIFT**	**P_ref**	**P_alt**	**ΔLog**	**DynaMut ΔΔG**
Case 1	*TGFBR1*	R487Q	31.00	0.99	0.90	0.00	1.00	8.02E-05	-9.43	-0.63
Case 2	*TGFBR2*	E555K	27.10	0.51	0.50	3.00E-03	1.00	2.60E-04	-8.22	0.23
Case 3	*TGFBR2*	E428D	22.50	1.00	0.92	0.00	0.95	7.60E-05	-9.42	-1.04
Case 4	*TGFBR2*	I218V	22.30	0.07	0.31	0.18	0.99	2.53E-03	-5.97	-0.10
Case 5	*TGFB2*	H2R	21.40	0.07	0.25	4.00E-03	0.34	0.16	-0.74	-0.04
Case 6	*TGFB2*	D215E	14.63	0.14	0.13	1.00	0.94	2.88E-03	-5.78	-0.54
Case 7	*TGFB2*	H208Q	0.20	0.08	0.10	0.06	0.67	0.01	-4.18	0.09
Case 8	*SMAD3*	L153Q	29.50	0.39	0.86	0.02	1.00	6.92E-05	-9.58	-0.12

Supplementary Table S2 presents functional prediction scores, local sequence-context metrics, and structural stability estimates for the same eight primary variants. Columns report CADD, AlphaMissense pathogenicity probability, REVEL, SIFT, reference and alternate amino-acid probabilities (P_ref and P_alt), log-odds change (ΔLog), and DynaMut predicted change in free energy of folding (ΔΔG, $kcal \cdot mol^{-1}$). Higher pathogenic/deleterious/destabilising signals are shaded pink/red, intermediate values yellow, and lower or benign signals green. Thresholds: CADD ≥20 pink, 15–19.99 yellow, <15 green; AlphaMissense ≥0.80 pink, 0.34–0.79 yellow, <0.34 green; REVEL ≥0.75 pink, 0.50–0.74 yellow, <0.50 green; SIFT <0.05 pink, 0.05–0.10 yellow, >0.10 green; P_ref ≥0.90 pink, 0.50–0.89 yellow, <0.50 green; P_alt <0.01 pink, 0.01–0.09 yellow, ≥0.10 green; ΔLog <−3 pink, −3 to −1 yellow, ≥−1 green; DynaMut ΔΔG ≤−0.50 pink, −0.50 to 0 yellow, ≥0 green. AlphaMissense cell-shading bands (≥0.80 / 0.34–0.79 / <0.34) are visualization bins only and are distinct from the CADD × AlphaMissense quadrant threshold of 0.564 used in Supplementary Figure S1 and Supplementary Table S3.

Supplementary Table S3. Evidence triangulation with phenotype–variant concordance grades across eight Florida cases.

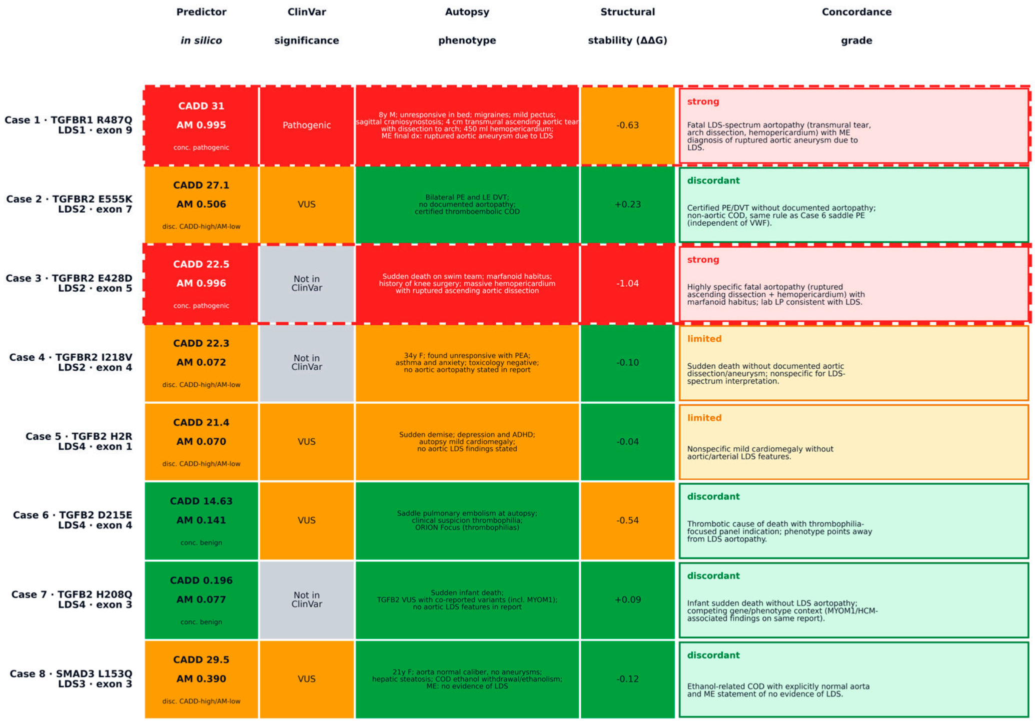

Heatmap of pathogenicity support across four evidence axes for Cases 1–8: (1) in silico predictors (CADD and AlphaMissense concordance quadrant; thresholds CADD ≥ 20.0 and AlphaMissense ≥ 0.564), (2) ClinVar clinical significance bucket, (3) phenotype–variant concordance grade for LDS/FTAAD-spectrum aortopathy interpretation, and (4) structural stability (DynaMut predicted ΔΔG; not FoldX; not experimental structure). The right-hand column shows the autopsy-led concordance grade. Case 2 (PE/DVT without aortopathy; TGFBR2 VUS) is graded discordant under the same rule as Case 6 (saddle PE). Series counts: strong n = 2 (Cases 1 and 3); limited n = 2 (Cases 4 and 5); discordant n = 4 (Cases 2, 6, 7, and 8); not assessable n = 0. In silico quadrants are independent of autopsy grades and do not override them. Axis colors encode computational or ClinVar support and are not themselves concordance grades.

Supplementary Table S4. Autopsy-context fit of secondary (additional/CNV) variants reported alongside the eight primary alleles.

Supplementary — additional / CNV variants: incidental/autopsy fit (PROPOSED)

	Predictor *in silico*	Lab classification	Autopsy phenotype	Incidental/autopsy fit of secondary variant
**Case 1 (no additional variants)**	N/A	N/A	8y M; unresponsive in bed; migraines; mild pectus; sagittal craniosynostosis; 4 cm transmural ascending aortic tear with dissection to arch; 450 ml hemopericardium; ME final dx: ruptured aortic aneurysm due to LDS	n/a — no additional variants<br>C1 report has no Additional Variants / CNV table (primary allele only on the summary row).
**Case 2 · ALPK3 T918I het Cardiomyopathy, familial hypertrophic 27**	**CADD 15.24 AM 0.146** conc. benign	VUS	Bilateral PE and LE DVT; clinical suspicion thrombophilia; no aortic LDS features in report	**incidental (PROPOSED)**<br>AR cardiomyopathy VUS het; no cardiac COD stated (PE/DVT).
**Case 2 · COL4A3 V11M het COL4A3 related disorder**	**CADD 15.01 AM 0.277** conc. benign	VUS	Bilateral PE and LE DVT; clinical suspicion thrombophilia; no aortic LDS features in report	**incidental (PROPOSED)**<br>COL4A3 VUS (basement-membrane spectrum); unrelated to PE/DVT COD.
**Case 2 · GCH1 c.626+2dup het Dystonia, DOPA-responsive, Hyperphenylala...**	N/A	Likely pathogenic	Bilateral PE and LE DVT; clinical suspicion thrombophilia; no aortic LDS features in report	**incidental (PROPOSED)**<br>GCH1 LP (dystonia/BH4); unrelated to bilateral PE/DVT.
**Case 2 · HBB E7V het HBB related disorder**	**CADD 14.4 AM 0.223** conc. benign	Pathogenic	Bilateral PE and LE DVT; clinical suspicion thrombophilia; no aortic LDS features in report	**incidental (PROPOSED)**<br>Pathogenic HBB het = sickle-cell carrier; incidental to PE/DVT.
**Case 2 · MAST2 R1086W het MAST2 related disorder**	**CADD 28.6 AM 0.918** conc. pathogenic	VUS	Bilateral PE and LE DVT; clinical suspicion thrombophilia; no aortic LDS features in report	**uncertain (PROPOSED)**<br>AD VUS in poorly defined MAST2 disorder; no clear PE/DVT mechanism.
**Case 2 · MMACHC E106K het Methylmalonic aciduria and homocystinuria...**	**CADD 28.4 AM 0.565** conc. pathogenic	VUS	Bilateral PE and LE DVT; clinical suspicion thrombophilia; no aortic LDS features in report	**incidental (PROPOSED)**<br>AR cblC VUS het without second allele; unrelated to PE/DVT.
**Case 2 · VWF CNV del · CNV het VWF related disorder**	N/A	Likely pathogenic	Bilateral PE and LE DVT; clinical suspicion thrombophilia; no aortic LDS features in report	**uncertain (PROPOSED)**<br>Competing VWF CNV (hemostasis-gene context only); VWD/thrombophilia not established.
**Case 2 · VWF R2507W het VWF related disorder**	**CADD 21.1 AM 0.083** disc. CADD↑/AM↓	VUS	Bilateral PE and LE DVT; clinical suspicion thrombophilia; no aortic LDS features in report	**uncertain (PROPOSED)**<br>Co-reported VWF missense VUS; autopsy fit uncertain; not a VWD/thrombophilia diagnosis.
**Case 3 · GFM1 c.689+908G>A het Combined oxidative phosphorylation defici...**	**CADD 5.082 AM N/A** CADD-low AM N/A	Likely pathogenic	Sudden death on swim team; marfanoid habitus; history of knee surgery; massive hemopericardium with ruptured ascending aortic dissection	**incidental (PROPOSED)**<br>GFM1 LP AR het (oxphos); does not explain ruptured aortic dissection.
**Case 3 · MVK N205D het MVK related disorder**	**CADD 28.2 AM 0.862** conc. pathogenic	Pathogenic	Sudden death on swim team; marfanoid habitus; history of knee surgery; massive hemopericardium with ruptured ascending aortic dissection	**incidental (PROPOSED)**<br>Pathogenic MVK het = AR carrier; incidental to fatal aortopathy.
**Case 3 · TMEM260 V39A het Structural heart defects and renal anomal...**	**CADD 21.9 AM 0.177** disc. CADD↑/AM↓	VUS	Sudden death on swim team; marfanoid habitus; history of knee surgery; massive hemopericardium with ruptured ascending aortic dissection	**incidental (PROPOSED)**<br>TMEM260 VUS AR het; not a fit for acute ascending dissection COD.
**Case 3 · TRPM4 V912I het Progressive familial heart block, type IB...**	**CADD 20.5 AM 0.111** disc. CADD↑/AM↓	VUS	Sudden death on swim team; marfanoid habitus; history of knee surgery; massive hemopericardium with ruptured ascending aortic dissection	**incidental (PROPOSED)**<br>TRPM4 VUS (heart-block spectrum); aortic dissection explained by the primary allele.
**Case 4 · C1QA P125Qfs het C1q deficiency 1**	N/A	Likely pathogenic	34y F; found unresponsive with PEA; asthma and anxiety; toxicology negative; no aortic aortopathy stated in report	**incidental (PROPOSED)**<br>C1QA LP AR het (complement deficiency); unrelated to PEA COD.
**Case 4 · MYOM1 T215_S220dup het MYOM1- Related Disorder**	N/A	VUS	34y F; found unresponsive with PEA; asthma and anxiety; toxicology negative; no aortic aortopathy stated in report	**uncertain (PROPOSED)**<br>MYOM1 VUS (HCM-associated) without stated cardiac phenotype on report.
**Case 4 · NEXN P244S het NEXN- Related Disorder**	**CADD 22.2 AM 0.176** disc. CADD↑/AM↓	VUS	34y F; found unresponsive with PEA; asthma and anxiety; toxicology negative; no aortic aortopathy stated in report	**uncertain (PROPOSED)**<br>NEXN VUS (cardiomyopathy gene) vs PEA SD without documented cardiomyopathy.
**Case 4 · QARS1 R463X het Microcephaly, progressive, seizures, and...**	**CADD 38.0 AM N/A** CADD-high AM N/A	Pathogenic	34y F; found unresponsive with PEA; asthma and anxiety; toxicology negative; no aortic aortopathy stated in report	**incidental (PROPOSED)**<br>Pathogenic QARS1 AR het (neurodevelopmental); incidental carrier.
**Case 5 · BBS1 M390R het Bardet-Biedl syndrome 1**	**CADD 25.3 AM 0.847** conc. pathogenic	Pathogenic	Sudden demise; depression and ADHD; autopsy mild cardiomegaly; no aortic LDS findings stated	**incidental (PROPOSED)**<br>Pathogenic BBS1 AR het; incidental carrier vs mild cardiomegaly SD.
**Case 5 · GAA c.-32-13T>G het Glycogen storage disease II**	**CADD 2.897 AM N/A** CADD-low AM N/A	Pathogenic	Sudden demise; depression and ADHD; autopsy mild cardiomegaly; no aortic LDS findings stated	**incidental (PROPOSED)**<br>Pathogenic GAA het = Pompe carrier; AR disease not confirmed.
**Case 5 · RNASEH2B A177T het Aicardi-Goutieres syndrome 2**	**CADD 20.5 AM 0.116** disc. CADD↑/AM↓	Pathogenic	Sudden demise; depression and ADHD; autopsy mild cardiomegaly; no aortic LDS findings stated	**incidental (PROPOSED)**<br>Pathogenic RNASEH2B AR het (AGS); incidental to nonspecific SD.
**Case 5 · SLC22A5 L202P het Carnitine deficiency, systemic primary**	**CADD 28.0 AM 0.549** disc. CADD↑/AM↓	VUS	Sudden demise; depression and ADHD; autopsy mild cardiomegaly; no aortic LDS findings stated	**incidental (PROPOSED)**<br>SLC22A5 VUS AR het (carnitine); no confirmed metabolic COD.
**Case 6 · MCFD2 c.149+5G>A het Factor V and factor VIII, combined defici...**	**CADD 23.7 AM N/A** CADD-high AM N/A	Likely pathogenic	Saddle pulmonary embolism at autopsy; clinical suspicion thrombophilia; ORION Focus (thrombophilias)	**uncertain (PROPOSED)**<br>MCFD2 LP on thrombophilia panel, but AR F5F8D is bleeding; no 2nd allele.
**Case 6 · PCCB R514X het Propionicacidemia**	**CADD 39.0 AM N/A** CADD-high AM N/A	Pathogenic	Saddle pulmonary embolism at autopsy; clinical suspicion thrombophilia; ORION Focus (thrombophilias)	**incidental (PROPOSED)**<br>Pathogenic PCCB AR het (propionic acidemia); incidental to saddle PE.
**Case 7 · ABCC6 C606S het ABCC6 related disorder**	**CADD 14.78 AM 0.110** conc. benign	VUS	Sudden infant death; TGFB2 VUS with co-reported variants (incl. MYOM1); no aortic LDS features in report	**incidental (PROPOSED)**<br>ABCC6 VUS (PXE spectrum); does not explain sudden infant death.
**Case 7 · ASL R379C het Argininosuccinic aciduria**	**CADD 32.0 AM 0.243** disc. CADD↑/AM↓	Pathogenic	Sudden infant death; TGFB2 VUS with co-reported variants (incl. MYOM1); no aortic LDS features in report	**incidental (PROPOSED)**<br>Pathogenic ASL AR het (urea-cycle); incidental carrier.
**Case 7 · ELN G649A het Supravalvar aortic stenosis**	**CADD 11.32 AM 0.133** conc. benign	VUS	Sudden infant death; TGFB2 VUS with co-reported variants (incl. MYOM1); no aortic LDS features in report	**incidental (PROPOSED)**<br>ELN VUS (SVAS) without stated aortic/vascular infant phenotype.
**Case 7 · GATA5 R228Q het Congenital heart defects, multiple types,...**	**CADD 23.4 AM 0.124** disc. CADD↑/AM↓	VUS	Sudden infant death; TGFB2 VUS with co-reported variants (incl. MYOM1); no aortic LDS features in report	**uncertain (PROPOSED)**<br>GATA5 VUS (CHD spectrum) vs SIDS without documented congenital heart defect.
**Case 7 · HBB E7V het Sickle cell disease**	**CADD 14.4 AM 0.223** conc. benign	Pathogenic	Sudden infant death; TGFB2 VUS with co-reported variants (incl. MYOM1); no aortic LDS features in report	**incidental (PROPOSED)**<br>Pathogenic HBB het = sickle-cell carrier; incidental to SIDS.
**Case 7 · INSR T1187M het INSR related disorder**	**CADD 27.2 AM 0.924** conc. pathogenic	VUS	Sudden infant death; TGFB2 VUS with co-reported variants (incl. MYOM1); no aortic LDS features in report	**incidental (PROPOSED)**<br>INSR VUS; unrelated to sudden infant death COD.
**Case 7 · MYOM1 R1067W het MYOM1 related disorder**	**CADD 22.4 AM 0.586** conc. pathogenic	VUS	Sudden infant death; TGFB2 VUS with co-reported variants (incl. MYOM1); no aortic LDS features in report	**uncertain (PROPOSED)**<br>MYOM1 VUS (HCM-associated) may compete with the primary allele for cardiac SD; phenotype undocumented.
**Case 7 · POGLUT1 R98W het POGLUT1 related disorder**	**CADD 25.6 AM 0.952** conc. pathogenic	Likely pathogenic	Sudden infant death; TGFB2 VUS with co-reported variants (incl. MYOM1); no aortic LDS features in report	**incidental (PROPOSED)**<br>POGLUT1 LP (MD/skin spectrum); unrelated to sudden infant death.
**Case 7 · TWNK Y428F het TWNK related disorder**	**CADD 24.4 AM 0.101** disc. CADD↑/AM↓	VUS	Sudden infant death; TGFB2 VUS with co-reported variants (incl. MYOM1); no aortic LDS features in report	**incidental (PROPOSED)**<br>TWNK VUS (mtDNA maintenance); no supporting metabolic/mito phenotype stated.
**Case 8 · TMEM94 c.3244-2A>T het Intellectual developmental disorder with...**	**CADD 27.9 AM N/A** CADD-high AM N/A	VUS	21y F; aorta normal caliber, no aneurysms; hepatic steatosis; COD ethanol withdrawal/ethanolism; ME: no evidence of LDS	**incidental (PROPOSED)**<br>TMEM94 VUS AR het; does not explain ethanol-related COD / normal aorta.

This table inventories additional/secondary variants (additional and CNV tiers) from the same postmortem molecular reports as the eight primary LDS-spectrum missense alleles (source: Supplementary Table S7 all-variants inventory). Primary LDS-spectrum variants are excluded. Case 1 has no Additional Variants or CNV section on the source report and is represented by a single note row. The Case 2 heterozygous likely pathogenic VWF exon deletion and co-reported VWF missense VUS are labeled as competing hemostasis-

gene context / uncertain autopsy fit only; they do not establish von Willebrand disease or a defined thrombophilia and do not change the primary TGFBR2 concordance grade (discordant).

Supplementary Table S5. Broad LDS/FTAAD phenotype feature review across eight Florida medical examiner cases.

Supplementary Table S5. Broad LDS/FTAAD phenotype checklist (Florida ME series, n = 8)

Feature	Case 1	Case 2	Case 3	Case 4	Case 5	Case 6	Case 7	Case 8
Aortic dilatation/aneurysm	Present	Not documented	Not documented	Not documented	Not documented	Not documented	Not documented	Absent
Aortic dissection/rupture	Present	Not documented	Present	Not documented	Not documented	Not documented	Not documented	Absent
Other arterial disease	Not documented	Not documented	Not documented	Not documented	Not documented	Not documented	Not documented	Not documented
Arterial tortuosity	Not documented	Not documented	Not documented	Not documented	Not documented	Not documented	Not documented	Not documented
Craniofacial features	Present	Not documented	Not documented	Absent	Not documented	Not documented	Not documented	Not documented
Skeletal features	Present	Not documented	Present	Not documented	Not documented	Not documented	Not documented	Not documented
Cutaneous features	Not documented	Not documented	Not documented	Not documented	Not documented	Not documented	Not documented	Not documented
Cardiac findings	Present	Not documented	Present	Not documented	Present	Not documented	Not documented	Not documented
Pulmonary findings	Not documented	Present	Not documented	Present	Not documented	Present	Not documented	Not documented
Allergic/inflammatory features	Not documented	Not documented	Not documented	Present	Not documented	Not documented	Not documented	Not documented
Family history	Not assessable	Not assessable	Not assessable	Not assessable	Not assessable	Not assessable	Not assessable	Not assessable

Present Absent Not documented Not assessable

Case-by-case scoring of broad Loeys–Dietz / FTAAD-spectrum features adapted from GeneReviews clinical descriptions for Cases 1–8. Rows include aortic dilatation/aneurysm, aortic dissection/rupture, other arterial disease, arterial tortuosity, craniofacial features, skeletal features, cutaneous features, cardiac findings, pulmonary findings, allergic/inflammatory features when assessable, and family history. Cells are scored Present, Absent, Not documented, or Not assessable. Present requires explicit documentation in medical examiner notes, laboratory extractions, or autopsy narrative; Absent requires an explicit negative statement (for example, Case 8 normal-caliber aorta without aneurysms); Not documented is the default when a feature is unmentioned; family history is Not assessable for all cases after de-identification. Sudden death alone is not scored as an LDS feature. No gene lists, causes of death, or ACMG classifications are invented for this checklist.

Supplementary Table S6. Per-case molecular testing, interpretation scope, coverage, and CNV documentation

Legend. All eight cases underwent whole-exome sequencing. Reports differed in downstream interpretation strategy: some used phenotype-driven prioritization of genes from the exome data, whereas others restricted interpretation and reporting to a predefined indication-specific gene set. Clinical indication, gene-analysis scope, coverage information, sequencing metrics, and CNV findings were abstracted directly from the available laboratory reports and requisitions. Information not documented in the available records is reported as such rather than inferred.

Case	Clinical indication / presentation documented in report	Sequencing assay	Downstream interpretation strategy	Documented gene-analysis scope	Coverage / sequencing metrics documented	CNV result / reporting	Primary LDS-spectrum finding
1	History of migraines; HPO term: Migraine	Whole-exome sequencing (WES)	Phenotype-/indication-driven interpretation of exome data	77 genes explicitly listed	75/77 genes at 100% coverage; PMS2 98.33%; TNXB 92.68%; ~11.59 Gb generated	No explicit case-level CNV result identified	TGFBR1 c.1460G>A (p.Arg487Gln) - Pathogenic
2	Autopsy: bilateral pulmonary emboli with lower-extremity DVT; clinical suspicion: thrombophilia	WES	Interpretation restricted to a predefined indication-specific gene set	Thrombophilia-focused gene set; complete enumerated list not recovered from supplied report	~11.31 Gb generated; 10.55 Gb passed mapping-quality cutoff	Positive: heterozygous likely pathogenic intragenic VWF deletion reported; report also contains a generic negative-CNV statement	TGFBR2 c.1663G>A (p.Glu555Lys) - VUS
3	Sudden death during swim practice; marfanoid habitus; autopsy showed massive hemopericardium with ruptured ascending aortic dissection; suspicion of connective-tissue disorder / Marfan / LDS	WES	Phenotype-driven interpretation of exome data	No complete analyzed gene list recovered	~16.87 Gb generated; 16.35 Gb passed mapping-quality cutoff	No clinically significant CNVs detected	TGFBR2 c.1284A>T (p.Glu428Asp) - Likely pathogenic
4	Found unresponsive in bed, pulseless with PEA; history of asthma and anxiety	WES	Phenotype-driven interpretation of exome data	No complete phenotype-driven gene list recovered	~9.01 Gb generated; 8.58 Gb passed mapping-quality cutoff	No clinically significant CNVs detected	TGFBR2 c.652A>G (p.Ile218Val) - VUS
5	Sudden demise; history of depression/ADHD; autopsy: mild cardiomegaly	WES	Phenotype-driven interpretation of exome data	No complete analyzed gene list recovered	~11.08 Gb generated; 10.35 Gb passed mapping-quality cutoff	No explicit case-level CNV result identified	TGFB2 c.5A>G (p.His2Arg) - VUS
6	Saddle embolism at autopsy; clinical suspicion: thrombophilia	WES	Interpretation restricted to a predefined indication-specific gene set	46 thrombophilia-associated genes explicitly listed	All 46 genes reported at 100% coverage; ~8.89 Gb generated; 8.20 Gb passed mapping-quality cutoff	No explicit case-level CNV result identified	TGFB2 c.645C>G (p.Asp215Glu) - VUS
7	Sudden infant death	WES	Phenotype-driven interpretation of exome data	No complete analyzed gene list recovered	~9.67 Gb generated; 9.33 Gb passed mapping-quality cutoff	No clinically significant CNVs detected	TGFB2 c.624T>A (p.His208Gln) - VUS
8	Sudden death	WES	Phenotype-driven interpretation of exome data	No complete analyzed gene list recovered	~20.73 Gb generated; 20.21 Gb passed mapping-quality cutoff	No clinically significant CNVs detected	SMAD3 c.458T>A (p.Leu153Gln) - VUS

Note on Case 2 CNV reporting. The report explicitly describes a likely pathogenic intragenic VWF deletion but also contains a generic statement that no clinically significant CNVs were detected; both statements are retained to reflect the source report.

Supplementary Table S7. Complete laboratory-reported variant inventory (n = 40 rows) for eight Florida medical examiner decedents.

Full inventory moved from former main-text Table 2: 8 primary LDS/FTAAD-spectrum alleles, 31 additional findings, and 1 CNV. Case 2 primary concordance is discordant. The Case 2 VWF CNV is competing hemostasis-gene context only (VWD/thrombophilia not established). Main-text Table 2 is the slim subset (primaries plus Case 2 VWF CNV and Case 7 MYOM1).

Case	Primary autopsy finding	Gene	HGVS	Zyg.	Lab class	Role	Concordance / incidental
1	Ruptured ascending aortic tear/dissection; hemopericardium	TGFBR1	c.1460G>A (p.Arg487Gln)	Het	P	primary	strong
2	Bilateral PE + LE DVT	TGFBR2	c.1663G>A (p.Glu555Lys)	Het	VUS	primary	discordant
	Bilateral PE + LE DVT	VWF	12:g.(?_6127521)_(6134874_?)del	Het	LP	CNV	competing VWF CNV; VWD/thrombophilia not established
	Bilateral PE + LE DVT	MAST2	c.3256C>T (p.Arg1086Trp)	Het	VUS	additional	uncertain
	Bilateral PE + LE DVT	VWF	c.7519C>T (p.Arg2507Trp)	Het	VUS	additional	uncertain
	Bilateral PE + LE DVT	COL4A3	c.31G>A (p.Val11Met)	Het	VUS	additional	incidental
	Bilateral PE + LE DVT	GCH1	c.626+2dup	Het	LP	additional	incidental
	Bilateral PE + LE DVT	HBB	c.20A>T (p.Glu7Val)	Het	P	additional	incidental
	Bilateral PE + LE DVT	MMACHC	c.316G>A (p.Glu106Lys)	Het	VUS	additional	incidental
	Bilateral PE + LE DVT	ALPK3	c.2753C>T (p.Thr918Ile)	Het	VUS	additional	incidental
3	Ruptured ascending aortic dissection; hemopericardium	TGFBR2	c.1284A>T (p.Glu428Asp)	Het	LP	primary	strong
	Ruptured ascending aortic dissection; hemopericardium	TRPM4	c.2734G>A (p.Val912Ile)	Het	VUS	additional	incidental
	Ruptured ascending aortic dissection; hemopericardium	MVK	c.613A>G (p.Asn205Asp)	Het	P	additional	incidental
	Ruptured ascending aortic dissection; hemopericardium	GFM1	c.689+908G>A	Het	LP	additional	incidental
	Ruptured ascending aortic dissection; hemopericardium	TMEM260	c.116T>C (p.Val39Ala)	Het	VUS	additional	incidental

4	Sudden death (PEA); COD indeterminate	TGFBR2	c.652A>G (p.Ile218Val)	Het	VUS	primary	limited
	Sudden death (PEA); COD indeterminate	NEXN	c.730C>T (p.Pro244Ser)	Het	VUS	additional	uncertain
	Sudden death (PEA); COD indeterminate	MYOM1	c.642_659dup (p.Thr215_Ser220dup)	Het	VUS	additional	uncertain
	Sudden death (PEA); COD indeterminate	QARS1	c.1387C>T (p.Arg463Ter)	Het	P	additional	incidental
	Sudden death (PEA); COD indeterminate	C1QA	c.374del (p.Pro125GlnfsTer157)	Het	LP	additional	incidental
5	Mild cardiomegaly; sudden demise	TGFB2	c.5A>G (p.His2Arg)	Het	VUS	primary	limited
	Mild cardiomegaly; sudden demise	GAA	c.-32-13T>G	Het	P	additional	incidental
	Mild cardiomegaly; sudden demise	RNASEH2B	c.529G>A (p.Ala177Thr)	Het	P	additional	incidental
	Mild cardiomegaly; sudden demise	BBS1	c.1169T>G (p.Met390Arg)	Het	P	additional	incidental
	Mild cardiomegaly; sudden demise	SLC22A5	c.605T>C (p.Leu202Pro)	Het	VUS	additional	incidental
6	Saddle pulmonary embolism	TGFB2	c.645C>G (p.Asp215Glu)	Het	VUS	primary	discordant
	Saddle pulmonary embolism	MCFD2	c.149+5G>A	Het	LP	additional	uncertain
	Saddle pulmonary embolism	PCCB	c.1540C>T (p.Arg514Ter)	Het	P	additional	incidental
7	Sudden infant death	TGFB2	c.624T>A (p.His208Gln)	Het	VUS	primary	discordant
	Sudden infant death	ABCC6	c.1817G>C (p.Cys606Ser)	Het	VUS	additional	incidental
	Sudden infant death	MYOM1	c.3199C>T (p.Arg1067Trp)	Het	VUS	additional	uncertain
	Sudden infant death	ELN	c.1946G>C (p.Gly649Ala)	Het	VUS	additional	incidental
	Sudden infant death	GATA5	c.683G>A (p.Arg228Gln)	Het	VUS	additional	uncertain
	Sudden infant death	HBB	c.20A>T (p.Glu7Val)	Het	P	additional	incidental

	Sudden infant death	ASL	c.1135C>T (p.Arg379Cys)	Het	P	additional	incidental
	Sudden infant death	POGLUT1	c.292C>T (p.Arg98Trp)	Het	LP	additional	incidental
	Sudden infant death	TWNK	c.1283A>T (p.Tyr428Phe)	Het	VUS	additional	incidental
	Sudden infant death	INSR	c.3560C>T (p.Thr1187Met)	Het	VUS	additional	incidental
8	Ethanol withdrawal; normal-caliber aorta	SMAD3	c.458T>A (p.Leu153Gln)	Het	VUS	primary	discordant
	Ethanol withdrawal; normal-caliber aorta	TMEM94	c.3244-2A>T	Het	VUS	additional	incidental
